# Handgrip strength and the GLIM criteria are markers for nutritional risk in patients treated in the SUS (Government Health System)

**DOI:** 10.3389/fnut.2025.1630142

**Published:** 2025-09-11

**Authors:** Vânia Aparecida Leandro-Merhi, Lucas Rosasco Mazzini, Larissa Silveira Stopiglia, Julia Pizzo Teixeira, Vitor Alexandre Camargo Barbieri, Rafael Iglesias Seccacci

**Affiliations:** ^1^Postgraduate Program in Health Sciences, School of Life Sciences, Pontifical Catholic University of Campinas, Campinas, Brazil; ^2^Scientific Initiation Scholarship, School of Medicine, School of Life Sciences, Pontifical Catholic University of Campinas, Campinas, Brazil; ^3^Scientific Initiation Scholarship, School of Nutrition, School of Life Sciences, Pontifical Catholic University of Campinas, Campinas, Brazil; ^4^Scientific Initiation Scholarship, School of Nutrition, School of Life Sciences, Pontifical Catholic University of Campinas, Campinas, Brazil; ^5^Scientific Initiation Scholarship, School of Medicine, Pontifical Catholic University of Campinas (PUC Campinas), School of Life Sciences, Campinas, Brazil; ^6^Scientific Initiation Scholarship, School of Medicine, Pontifical Catholic University of Campinas (PUC Campinas), School of Life Sciences, Campinas, Brazil

**Keywords:** nutritional status, hospitalized patients, subjective global assessment (SGA), nutritional risk screening-2002 (NRS), handgrip strength (HGS), Global Leadership Initiative on Malnutrition (GLIM) criteria

## Abstract

**Background:**

Although several tools are available for nutritional diagnosis, some difficulties may be encountered in the assessment of bedridden patients.

**Objective:**

To investigate the validity and feasibility of the Global Leadership Initiative on Malnutrition (GLIM) criteria and the handgrip strength (HGS) as markers of nutritional status in comparison with the Nutritional Risk Screening-2002 (NRS) and the Subjective Global Assessment (SGA) in patients hospitalized under the Government Health System.

**Method:**

Cross-sectional study conducted with patients in the surgical wards of a university hospital. Clinical and nutritional data were investigated using the GLIM criteria, HGS, NRS and SGA. The Chi-square or Fisher’s exact test, Mann–Whitney test, Kappa coefficient and simple and multiple logistic regression analysis were used for data analysis, with stepwise variable selection criteria and a significance level of 5%. The statistical software used for the analyses was the Statistical Analysis System (SAS).

**Results:**

A total of 160 patients were assessed; their mean age was 59.31 ± 16.14 years. Out of these, 62.5% (*n* = 100) were male and 37.5% (*n* = 60) female. By multiple regression analysis it was found that HGS *(p = 0.0327; OR = 0.956 (1.046); IC95% = 0.917; 0.996 (1.004; 1.091)* and the GLIM criteria *(p = <0.0001; OR = 26.381; IC95% = 9.996; 69.620)* were factors associated with nutritional risk according to the NRS. And only the GLIM criteria were the factor associated with malnutrition according to the SGA *(p = <0.0001; OR = 27.710; IC95% = 11.306; 67.916)*. Malnutrition according to the GLIM criteria presented 26.4 times more chance of nutritional risk according to the NRS and 27.71 times more chance of malnutrition according to the SGA.

**Conclusion:**

In patients treated under the SUS (Government Health System), the HGS and the GLIM criteria were markers for nutritional risk according to the NRS. It is suggested that those tools could be incorporated into the routine nutritional care in the hospital setting by the Government Health System.

## Introduction

The Guidelines of the European Society for Clinical Nutrition and Metabolism (ESPEN) have already set out how to screen patients who are at nutritional risk and to diagnose nutritional status in different clinical conditions; those guidelines also describe the thresholds for classifying malnutrition severity and the Global Leadership Initiative on Malnutrition (GLIM) criteria (1, 2) and handgrip strength ability to predict malnutrition in hospitalized patients. This is a recently developed and validated criterion for the diagnosis of malnutrition in adult patients (2–4), considering phenotypic and etiological criteria for this diagnosis (2–5).

A cohort study compared different nutritional screening tools with the GLIM criteria in hospitalized patients (3), showing a prevalence of malnutrition in 46% of the patients according to the GLIM criteria and GLIM criteria concordance of 89%, 53%, and 62% with the Malnutrition Universal Screening Tool (MUST), the Subjective Global Assessment (SGA) and the Nutritional Risk Screening-2002 (NRS), respectively. The study reported that all screening tools were of moderate value for diagnosing malnutrition (3). Another study also showed that all screening instruments were of moderate value for diagnosing malnutrition and that the nutritional status should be determined using the GLIM criteria (4). GLIM showed satisfactory validity for the diagnosis of malnutrition in the clinical practice in non-critically ill in-patients (5). In hospitalized older adults it was reported that the prevalence of malnutrition defined by the GLIM criteria could vary according to each tool used (6). In another observational and prospective study conducted with cancer patients (7), a high prevalence of malnutrition was observed, and it was also reported that SGA and the GLIM criteria, especially in conjunction with the handgrip strength (HGS), were useful tools for diagnosing malnutrition and presented a similar predictive value in relation to mortality (7).

An accessible tool that contributes to the diagnosis of malnutrition is the HGS, which is also an alternative for estimating muscle mass (8–11). HGS was reported in another study as being the most sensitive method to identify individuals at risk of malnutrition (12) and with regression models HGS was significantly and inversely associated with length of hospital stay, independently of multiple covariates, including age (13). Thus, HGS could be considered a reliable clinical method for nutritional assessment in different clinical situations (13, 14) and a good indicator of general muscle strength (9, 14, 15).

Although several tools are available for nutritional diagnosis, some difficulties may be encountered in the assessment of bedridden patients. There are still knowledge gaps regarding the assessment of the nutritional status of in-patients, especially those bedridden. In fully bedridden patients, additional tools to aid in such diagnosis are often needed, including objective and subjective methods. In the hospital services with a high volume of patients treated under the SUS (Government Health System) incorporating tools complementary to those already used in clinical practice could contribute to more effective decision-making in nutritional care. Furthermore, considering the prevalence of hospital malnutrition in Brazil (16) and in other countries (17), the early implementation of nutritional screening measures in the hospital stay, could have a positive impact reducing adverse clinical outcomes.

Thus, the purpose of this study was to investigate the validity and feasibility of the Global Leadership Initiative on Malnutrition (GLIM) criteria and handgrip strength (HGS) as markers of nutritional status in comparison with the NRS and SGA in patients hospitalized under the Government Health System (SUS).

## Method

### Study design and location

This was a cross-sectional study conducted with a population of low income patients admitted by the Government Health System (SUS) in the surgical wards of a university hospital. The study was designed according to the Strengthening the Reporting of Observational Studies in Epidemiology (STROBE) guidelines for cross-sectional studies (18).

### Study participants, inclusion, and exclusion criteria

Patients were recruited and invited to participate in the study at the beginning of their hospital stay. They received detailed instructions about the study and their participation materialized after their agreement and signing of the free and informed consent form (FICF). Patients admitted exclusively under the Government Health System (SUS) for different clinical and surgical conditions were considered eligible. The inclusion criteria were: be over 18 years of age; ability to understand and respond to the information provided; preserved motor and cognitive functions and having signed the FICF. The exclusion criteria were: patients with edema or ascites; patients hospitalized in isolation; in a critical terminal condition; with any type of dementia or Parkinson’s; with fractures of the upper or lower limbs and hospitalized for less than 48 h.

### Sample calculation

The sample size was calculated based on estimates of hospital malnutrition prevalence (16, 17), which can vary from 15% to 60%. For a prevalence of 60%, with a significance level of 5% and a sampling error of 8%, the number calculated was 145. We beefed up this sample by 10% (*n* = 160 patients) to compensate for possible data loss.

### Data collection

Data were collected directly from the medical records by the investigator in charge during patient care in the wards. Data were extracted from the electronic patient records in the hospital system after the patient was included in the study and/or retrospectively. Nutritional screening assessments and muscle function measurements were performed entirely by the investigator in charge, with the assistance of properly trained students, holders of scientific initiation scholarships.

### Variables studied

#### Explanatory variables (independent variables)

##### Global Leadership Initiative on Malnutrition (GLIM) criteria

According to specific methodology and recommendations from ESPEN, the GLIM criteria were determined considering the first stage of nutritional screening and the presence of at least one phenotypic criterion *(unintentional weight loss or low body mass index or reduction in muscle mass using arm circumference or calf circumference measurements)* and one etiological criterion *(severity of the disease or reduction of food intake)* (2–4). These criteria, in association or not with other nutritional markers, have recently been investigated with regard to their application in hospitalized patients (7, 19–22).

##### Handgrip strength (HGS)

HGS was assessed using a calibrated and validated Jamar hydraulic handgrip dynamometer, according to the protocols. Participants were instructed to sit, as has been proposed in clinical practice (9, 13, 14, 23, 24), with their shoulders pressed, elbow flexed at 90° and forearm and wrist in a neutral position (9); they were instructed to perform three maximum fingers compressions in each hand with brief pauses between measurements. The mean value of the three measurements was taken into account (9, 13, 14, 23). HGS was classified and compared with the cutoff values recommended by the revised European consensus on sarcopenia *(men < 27 kg, women < 16 kg)* (15). All patients were assessed with the same dynamometer model. All assessments were carried out by the investigator in charge.

#### Outcome variables

##### Nutritional diagnosis expressed by Nutritional Risk Screening-2002 (NRS)

The NRS was evaluated to determine the nutritional risk according to previously described recommendations (25, 26), considering the severity of the disease, decreased food consumption, weight loss, body mass index and the adjusted factor for individuals ≥ 70 years of age. The total score allowed the patient to be classified “at nutritional risk” *(score ≥ 3)* and without “nutritional risk” *(score < 3)* (25, 26).

##### Nutritional diagnosis expressed by Subjective Global Assessment (SGA)

SGA was assessed based on scores obtained from the history of body weight loss, dietary changes, gastrointestinal symptoms, functional capacity, diagnosis and physical examination, allowing the patient to be classified as well-nourished (eutrophic), mildly, moderately or severely malnourished (27, 28).

#### Control variables

Age, gender, and type of disease.

### Statistical analysis

In order to describe the characteristics of the sample, categorical variables frequency tables were developed; they included absolute frequency *(n)*, rate*(%)* values, and descriptive measures *(mean, standard deviation and median)* for quantitative variables. The chi-square test or Fisher’s exact test was used to compare proportions when necessary (between gender, disease and GLIM with NRS and SGA); the Mann–Whitney test was applied to compare continuous measures between two groups (between age and HGS with NRS and SGA). The Kappa coefficient was used to assess agreement between the GLIM criteria and the HGS with the NRS and SGA. Subsequently, simple and multiple logistic regression analyses were used to assess the factors that discriminated the outcomes of interest. The stepwise variable selection criterion was applied. The significance level adopted for the statistical tests was 5%. There was no correction for multiple comparisons. The statistical software used for the analyses was the Statistical Analysis System (SAS).

## Results

We evaluated 160 patients admitted to a surgical ward; their mean age was 59.31 ± 16.14 years *(median = 61 years);* a total 62.5% (*n* = 100) was male and 37.5% (*n* = 60) female; the disorders most frequently found were heart disease (*n* = 40, 25%), neoplasms (*n* = 40, 25%), kidney disease (*n* = 25, 15.6%), vascular disease (*n* = 23, 14.4%), lung disease (*n* = 12, 7.5%), orthopedic disease (*n* = 8, 5%), rheumatological disease (*n* = 5, 3.1%), and other illnesses (*n* = 7, 4.4%).

Regarding the instruments evaluated, 37.5% (*n* = 60) of the patients were classified as mildly malnourished by the SGA, 56.9% (*n* = 91) presented nutritional risk by the NRS and 44.4% (*n* = 71) were classified as malnourished according to the GLIM criteria. In our study, no patients were classified as moderately or severely malnourished by SGA. The mean HGS was 21.82 ± 10.22 kg *(median = 19.75)*. It was found that 57.5% (*n* = 92) of the patients assessed evidenced HGS values lower than the reference values recommended by the European consensus on sarcopenia (15).

### Descriptive analysis and comparisons of variables between the NRS instrument and the SGA

Table 1 shows a descriptive analysis and comparison of variables in relation to nutritional risk as classified by the NRS. There was a statistical difference in relation to age (*p* = 0.0010), type of disease (*p* = 0.0007), GLIM criteria (*p* < 0.0001), mean HGS (*p* = 0.0210) and HGS classified according to the recommended cutoff points (*p* = 0.0018). When comparing the variables in relation to the NRS, it was observed in the group of patients at nutritional risk, in older age individuals, with higher frequency of neoplasms, a greater number of patients classified as malnourished by the GLIM criteria *(71.4% in the group of patients with nutritional risk vs. 8.7% in the group of patients without nutritional risk)*, lower mean HGS values and a higher frequency of patients with HGS values below the reference standard *(68.1% in the group of patients with nutritional risk vs. 43.5% in the group of patients without nutritional risk)* (Table 1). In other words, patients with HGS classified below the reference standard and classified as malnourished according to the GLIM criteria presented a higher rate of nutritional risk by NRS.

**Table 1:** Descriptive analysis and comparisons of variables in the NRS instrument.

Variables studied	Category	Nutritional risk screening (NRS)		*p*-value
		With nutritional risk (*n* = 91)	Without nutritional risk (*n* = 69)	
Age (years)	X̅±SD	62.73 ± 16.33	54.81 ± 14.83	0.0010^a^
	mean	66.00	57.00	
mHGS	X̅±SD	19.90 ± 9.09	24.37 ± 11.11	0.0210^a^
	mean	18.67	21.83	
Gender	Female	34 (37.4%)	26 (37.7%)	0.9671^b^
	Male	57 (62.6%)	43 (62.3%)	
Disease	Cardiac	20 (22.0%)	20 (29.0%)	0.0007^c^
	Orthopedic	1 (1.1%)	7 (10.1%)	
	Pulmonary	9 (9.9%)	3 (4.3%)	
	Renal	12 (13.2%)	13 (18.8%)	
	Rheumatological	3 (3.3%)	2 (2.9%)	
	Vascular	11 (12.1%)	12 (17.4%)	
	Neoplasms	33 (36.3%)	7 (10.1%)	
	Other	2 (2.2%)	5 (7.2%)	
GLIM	Malnourished	65 (71.4%)	6 (8.7%)	<.0001^b^
	Not malnourished	26 (28.6%)	63 (91.3%)	
c mHGS	< reference	62 (68.1%)	30 (43.5%)	0.0018^a^
	≥ reference	29 (31.9%)	39 (56.5%)	

mHGS, mean handgrip strength; c mHGS, classified mean handgrip strength; GLIM: Global Leadership Initiative on Malnutrition criteria.

a Mann–Whitney test.

b Chi-square test.

c Fisher’s test.

Table 2 shows a descriptive analysis and comparison of variables in relation to the classification of nutritional status by SGA. There was a statistical difference in relation to age (*p* = 0.0006), type of disease (*p* = 0.0001), GLIM criteria (*p* < 0.0001) and HGS classified according to the recommended cutoff points (*p* = 0.0017). In the comparison of variables in relation to SGA; in the group of mildly malnourished patients, of older age individuals, with higher frequency of neoplasms, there were more patients classified as malnourished according to the GLIM criteria *(86.7% in the group of mildly malnourished patients vs. 19.0% in the group of well-nourished patients)* and higher frequency of patients with HGS values below the reference standard *(73.3% in the group of mildly malnourished patients vs. 48.0% in the group of well-nourished patients)* (Table 2). In other words, patients with HGS classified below the reference standard and classified as malnourished according to the GLIM criteria, were classified as being slightly malnourished according to the SGA.

**Table 2:** Descriptive analysis and comparisons of variables between the SGA instrument.

Variables studied	Category	Subjective global assessment (SGA)		*p*-value
		Well nourished (*n* = 100)	Slightly malnourished (*n* = 60)	
Age (years)	X̅±SD	56.32 ± 15.65	64.30 ± 15.82	0.0006^a^
	mean	58.00	67.50	
mHGS	X̅±SD	23.02 ± 10.29	19.83 ± 9.87	0.0789^a^
	mean	21.08	18.33	
Gender	Female	41 (41.0%)	19 (31.7%)	0.2378^b^
	Male	59 (59.0%)	41 (68.3%)	
Disease	Cardiac	33 (33.0%)	7 (11.7%)	0.0001^c^
	Orthopedic	8 (8.0%)	0 (0.0%)	
	Pulmonary	7 (7.0%)	5 (8.3%)	
	Renal	15 (15.0%)	10 (16.7%)	
	Rheumatological	2 (2.0%)	3 (5.0%)	
	Vascular	17 (17.0%)	6 (10.0%)	
	Neoplasms	13 (13.0%)	27 (45.0%)	
	Other	5 (5.0%)	2 (3.3%)	
GLIM	Malnourished	19 (19.0%)	52 (86.7%)	<.0001^b^
	Not malnourished	81 (81.0%)	8 (13.3%)	
c mHGS	<reference	48 (48.0%)	44 (73.3%)	0.0017^a^
	≥ reference	52 (52.0%)	16 (26.7%)	

mHGS, mean handgrip strength; c mHGS: classified mean handgrip strength; GLIM, Global Leadership Initiative on Malnutrition criteria.

a Mann–Whitney test.

b Chi-square test.

c Fisher’s test.

### Analysis of agreement between the GLIM criteria and the HGS with the NRS and the SGA

Table 3 shows the analysis of agreement between the GLIM criteria and the NRS and SGA instruments, using the Kappa coefficient. Moderate to good agreement was observed *[Kappa = 0.6061; 95%CI (0.4877; 0.7245)]* between the GLIM and the NRS, in the classification of patients considered at nutritional risk by the NRS and malnourished by the GLIM. Moderate to good agreement *[Kappa = 0.6527; 95%CI (0.5347; 0.7708)]* between the GLIM and the SGA was also evidenced in the classification of the patients evaluated. In the assessment of agreement between the HGS and the NRS and SGA instruments, using the Kappa coefficient (Table 4), we found a weak agreement between the HGS and the NRS *[Kappa = 0.2470; 95%CI (0.0957; 0.3982)] and between the HGS and the SGA (Kappa = 0.2289; 95%CI [0.0917; 0.3661]).*

**Table 3:** Analysis of agreement between the Global Leadership on Malnutrition (GLIM) criteria and the nutritional risk screening (NRS) and the subjective global assessment (SGA).

Global Leadership Initiative on Malnutrition (GLIM)	Nutritional risk screening (NRS)		Total
	With nutritional risk	Without nutritional risk	
Malnourished	65 (40.63%)	6 (3.75%)	71 (44.38%)
Not malnourished	26 (16.25%)	63 (39.38%)	89 (55.63%)
Total	91 (56.88%)	69 (43.13%)	160 (100.00%)
Kappa = 0.6061; IC95% (0.4877; 0.7245)			

Global Leadership Initiative on Malnutrition (GLIM)	Subjective global assessment (SGA)		Total
	Slightly malnourished	Well nourished	
Malnourished	52 (32.50%)	19 (11.88%)	71 (44.38%)
Not malnourished	8 (5.00%)	81 (50.63%)	89 (55.63%)
Total	60 (37.50%)	100 (62.50%)	160 (100.00%)
Kappa = 0.6527; 95%CI (0.5347; 0.7708)			

**Table 4:** Analysis of agreement between handgrip strength (HGS) and nutritional risk screening (NRS) and Subjective Global Assessment (SGA).

Handgrip strength (HGS)	Nutritional risk screening (NRS)		Total
	With nutritional risk	Without nutritional risk	
< reference	62 (38.75%)	30 (18.57%)	92 (57.50%)
≥ reference	29 (18.13%)	39 (24.38%)	68 (42.50%)
Total	91 (56.88%)	69 (43.13%)	160 (100.00%)
Kappa = 0.2470; 95%CI (0.0957; 0.3982)			
(HGS = men<27 kg, women<16 kg)^15^			

Handgrip strength (HGS)	Subjective Global Assessment (SGA)		Total
	Slightly malnourished	Well nourished	
< reference	44 (27.50%)	48 (30.00%)	92 (57.50%)
≥ reference	16 (10.00%)	52 (32.50%)	68 (42.50%)
Total	60 (37.50%)	100 (62.50%)	160 (100.00%)
Kappa = 0.2289; 95%CI (0.0917; 0.3661)			
(HGS = men <27 kg, women<16 kg)^15^			

### Study of the validity of the GLIM criteria and the HGS in relation to the NRS and the SGA, by univariate and multiple logistic regression

Table 5 shows the study of the validity of the GLIM criteria and the HGS in relation to the NRS, analyzed by univariate and multiple logistic regression. It was found that the HGS (*p = 0.0327; OR = 0.956 (1.046); 95%CI = 0.917; 0.996 (1.004; 1.091)* and the GLIM criteria *(p = <0.0001; OR = 26.381; 95%CI = 9.996; 69.620)* were discriminating factors of the nutritional risk classified by the NRS. Each unit less in the HGS increased the risk of malnutrition according to the NRS by 4.6%. Malnutrition by the GLIM criteria presented 26.4 times more chance of NRS malnutrition.

**Table 5:** Study of the validity of the GLIM criteria and the average HGS in relation to nutritional risk screening (NRS), analyzed by univariate and multiple logistic regression.

Variables	Category	*p*-value	OR	95% CI
Modeling the probability of NRS = At nutritional risk				
Univariate analysis				
Age		0.0027	1.032	1.011; 1.054
mHGS		0.0071	0.957	0.927; 0.988
Gender	male *vs.* female	0.9671	1.014	0.531; 1.934
Disease	Cardiac *vs.* other	0.3056	2.500	0.433; 14.430
	Orthopedic *vs.* other	0.4485	0.358	0.025; 5.111
	Pulmonary *vs.* other	0.0596	7.500	0.921; 61.047
	Renal *vs.* other	0.3673	2.308	0.375; 14.212
	Rheumatologic *vs.* other	0.2858	3.750	0.331; 42.467
	Vascular *vs.* other	0.3751	2.292	0.367; 14.323
	Neoplasm *vs.* other	0.0083	11.785	1.888; 73.571
GLIM	Malnourished *vs.* not malnourished	<0.0001	26.249	10.122; 68.071
c_mHGS	<ref. *vs.* ≥ref	0.0020	2.779	1.453; 5.317
Multiple analysis: stepwise selection process				
mHGS		0.0327	0.956 (1.046)	0.917; 0.996 (1.004; 1.091)
GLIM	Malnourished *vs.* not malnourished	<0.0001	26.381	9.996; 69.620

mHGS, mean handgrip strength; c mHGS, classified mean handgrip strength; ref, reference standard; GLIM, Global Leadership Initiative on Malnutrition criteria.

OR, odds ratio; 95% CI, 95% confidence interval.

Table 6 shows the study of the validity of the GLIM criteria and the HGS in relation to SGA, analyzed by univariate and multiple logistic regression. It was found that only the GLIM criteria constituted the independent discriminating factor for malnutrition classified by SGA *(p = <0.0001; OR = 27.710; 95%CI = 11.306; 67.916)*. When classified as being malnutrition by the GLIM criteria, the chance of presenting malnutrition by SGA was 27.710 times greater.

**Table 6:** Study of the validity of the GLIM criteria and the average HGS in relation to the subjective global assessment (SGA), analyzed by univariate and multiple logistic regression.

Variables	Category	*p*-value	OR	95% CI
Modeling the probability of SGA = With malnutrition				
Univariate analysis				
Age		0.0032	1.034	1.011; 1.058
mHGS		0.0580	0.969	0.937; 1.001
Gender	male *vs.* female	0.2389	1.500	0.764; 2.943
Disease	Cardiac *vs.* other	0.4973	0.530	0.085; 3.311
	Orthopedic *vs.* other	0.9762	–	
	Pulmonary *vs.* other	0.5702	1.786	0.241; 13.215
	Renal *vs.* other	0.5832	1.667	0.269; 10.334
	Rheumatologic *vs.* other	0.2858	3.750	0.331; 42.467
	Vascular *vs.* other	0.8965	0.882	0.134; 5.815
	Neoplasm *vs.* other	0.0679	5.192	0.886; 30.431
GLIM	Malnourished *vs.* not malnourished	<0.0001	27.710	11.306; 67.916
c_mHGS	<ref. *vs.* ≥ref	0.0020	2.979	1.489; 5.962
Multiple analysis: stepwise selection process				
GLIM	Malnourished *vs.* not malnourished	<0.0001	27.710	11.306; 67.916

mHGS: mean handgrip strength; c mHGS: classified mean handgrip strength; ref, reference standard; GLIM, Global Leadership Initiative on Malnutrition criteria.

OR, odds ratio; 95% CI, 95% confidence interval.

## Discussion

This study aimed to investigate the predictive capacity of the GLIM and HGS criteria for malnutrition in hospitalized adult patients, in comparison with other nutritional risk and malnutrition assessment instruments commonly used in the hospital setting.

Although several papers on this topic are available, there are still knowledge gaps regarding the type of population studied. It is important to emphasize that, unlike previous studies, our investigation was conducted with a population of lower income patients treated exclusively under the Government Health System (SUS). The hospital where this research was conducted is a large university hospital that serves a high flow of SUS patients it is a reference surgical hospital, with a large number of patients attended and surgeries.

The findings revealed that patients with HGS classified below the reference standard and malnourished according to the GLIM criteria were those with the highest rate of nutritional risk according to the NRS and the highest rate of malnutrition *(mildly malnourished)* according to the SGA. The data indicated moderate to good agreement between the GLIM and the NRS, and between the GLIM and the SGA. The same agreement was not found between the HGS and the NRS and SGA instruments. The fact that HGS is an objective assessment tool could explain the findings of low agreement with the subjective assessment instruments evaluated in the present study.

Through regression analysis, this investigation found that the GLIM criteria and the HGS agreed with the NRS results; when the patient’s nutritional status was classified as malnourished by the GLIM criteria and the chance of that patient being at nutritional risk by the NRS was high *(26.4 times higher)*. And the lower the value found in the HGS, the greater the chance of nutritional risk by the NRS. In the present study, potential confounding factors that could influence the odds ratio (OR) values, such as age, gender and type of disease, were considered. However, only two parameters remained significantly associated in the multiple model: HGS and the GLIM criteria. The high OR values (OR = 26) may have been influenced by the sample size (Table 1) and by the data distribution (considering that only six cases were diagnosed with malnutrition by the GLIM criteria but were not classified as at nutritional risk by the NRS).

These findings are in line with other studies in the literature indicating that the GLIM criteria and the NRS instrument were concordant methods for the diagnosis of nutritional risk and malnutrition in surgical patients (29). Another single-center observational study investigated the prevalence and risk of malnutrition with several assessment tools, which were also adopted in the present study (30). The authors reported that calf circumference, body weight, body mass index, phase angle and HGS were significantly lower in patients who presented nutritional risk by the NRS, malnutrition by the SGA and by the GLIM criteria (30). Another investigation in a prospective study that evaluated the GLIM criteria validity and the feasibility of use in the intensive care unit, patients showed that the GLIM criteria presented high sensitivity, moderate specificity and substantial agreement with the SGA in critically ill patients (31). The authors reported that malnutrition by the GLIM criteria was an independent predictor of extended ICU stay and readmission, and malnutrition by SGA increased the chances of readmission and the risk of death in the ICU (31).

In this study, in the regression analysis that assessed the feasibility of use of the GLIM criteria and HGS in relation to SGA, it was observed that only the GLIM criteria were discriminant for the classification of the nutritional condition by SGA. HGS was not associated with SGA. This result may be related to the fact that GLIM includes, in its assessment, aspects such as weight loss, reduced food intake, and severity of the disease, among other factors.

Findings similar to those of this study regarding the relationship between HGS and SGA showed 34% of malnourished patients by SGA and 37.2% of patients with reduced HGS, in addition to weak agreement for the diagnosis of malnutrition between HGS and SGA (32). Lower HGS was associated with prolonged hospital stay and death but was not accurate in the diagnosis of malnutrition during the hospitalization period (32). These findings corroborate the data of our study and suggest that further investigations ought to be carried out to deepen the feasibility of using the HGS measurements in hospitalized patients.

HGS has already been reported as a prognostic marker of mortality and an independent indicator of malnutrition in patients with acute decompensated heart failure in an investigation conducted with a sample size similar to that of this study (33). The authors reported 60% of malnutrition by SGA and lower HGS values in malnourished patients (33). The agreement between subjective and objective diagnostic methods has also been investigated, including the assessment of HGS in outpatients (34). The combination of several instruments for assessing malnutrition, such as the GLIM criteria, albumin, HGS and low muscle radiodensity, resulted in a model with high prognostic power for mortality (35). In another investigation carried out with in-patients, whose population was similar to that of our study, it was observed that HGS was more sensitive in identifying malnutrition when compared to other nutritional assessment indicators (12).

It is important to highlight that although the analysis between reduced HGS and the occurrence of unfavorable clinical outcomes was not the subject of investigation in our study although an investigation of such factors would be of great importance. In the study cited above, an association between reduced HGS and extended hospitalization stay and risk of death was observed, but without satisfactory accuracy in identifying malnutrition (32). Low HGS was observed as a simple and effective tool and predictor of all-cause and cardiovascular disease mortality (36), and it was also reported that in patients with low HGS, appetite and satiety should be monitored (37). In another study carried out with elderly patients hospitalized in surgical wards, HGS was not considered adequate for malnutrition screening (38), but the assessment of muscle health should be incorporated into nutritional care (10). A positive correlation between frailty and malnutrition was observed in a recent study of elderly surgical in-patients, which investigated the relationship between frailty and malnutrition and their effects on clinical outcomes (39). The authors reported a significant association between malnutrition and frailty and clinical outcomes (39).

The objective of our study was to explore the relationship between the GLIM and HGS criteria with the NRS and SGA instruments for the joint assessment of nutritional status and muscle strength. The data obtained suggest combining different instruments for the diagnosis of malnutrition together with muscle health markers (HGS) in the routine hospital clinical practice. However, it is important to note that these procedures may present different diagnoses according to the group of patients and the type of disease, and therefore they ought to be confirmed in future studies with the use of a larger sample.

Another aspect to be observed refers to the HGS cutoff points that were defined in this study based on the European consensus, which may not be fully representative of the Brazilian population. Further studies with this population profile are suggested to deepen our understanding of these issues.

Several nutritional diagnostic methodologies have already been used in routine nutritional care in hospitals. However, these methods are not always applied in an integrated and simultaneous manner. Often, only a portion of the available procedures are adopted, depending on the type of hospital and the number of healthcare professionals available for these activities. The HGS measurement and the GLIM criteria are easily applicable and feasible methods. However, their implementation depends on the availability of qualified professionals, adequate training, and additional costs involved in expanding multidisciplinary teams, especially within the Government Health System (SUS).

## Conclusion

In patients treated under the Government Health System, the HGS and the GLIM criteria were markers for nutritional risk according to the NRS. These tools could be incorporated into the routine nutritional care in the hospital practice of the Government Health System.

## Strengths and limitations of the study

The strengths of this study were demonstrated by the feasibility and validity of using all these instruments. Feasibility was assessed by the ease in obtaining these measures and tools and their application in bedridden patients. Validity was assessed by the relationship between the GLIM and HGS criteria with the other instruments (NRS and SGA). Another point is that 160 patients admitted exclusively to a surgical ward were included, and few of the eligible patients declined to participate in the study. It is important to emphasize that all protocols and guidelines for assessing GLIM and HGS were fully adhered to, according to the existing guidelines. Another relevant point to be highlighted is that there was no reliability variation between evaluators since the same researcher carried out all the evaluations. This study had no selection bias, as the excluded patients did not meet the clinical conditions required to participate. Exclusions were justified by factors such as the presence of any dementia or Parkinson’s disease, edema or ascites, fractures in the upper or lower limbs, hospitalization in isolation, critical or terminal clinical conditions, or hospital stay of less than 48 h.

As limitations, the sample size, the type of cross-sectional study, and the fact that the investigation was conducted in a single center could reduce a generalization of our study outcome. In addition, the HGS cutoff points adopted may not be appropriate for all populations. Because this study aimed to investigate and compare different nutritional diagnostic instruments, the associations between nutritional status and clinical outcomes, such as length of hospital stay, mortality, complications, and surgery, were not assessed. This limitation should be considered when interpreting the results. Therefore, future studies can explore these associations in greater depth.

Despite this, our study population was different, as this study was carried out with lower income patients hospitalized exclusively under the Government Health System, in other words a different casuistry with regard to previous works.

## Data Availability

The original contributions presented in the study are included in the article/supplementary material, further inquiries can be directed to the corresponding author.
